# The effect of exacerbations on lung density in α_1_-antitrypsin deficiency

**DOI:** 10.1183/23120541.00457-2022

**Published:** 2023-03-13

**Authors:** Charlie Strange, N. Gerard McElvaney, Claus F. Vogelmeier, Marcos Marin-Galiano, Michaela Buch-Haensel, Xiang Zhang, Younan Chen, Oliver Vit, Marion Wencker, Kenneth R. Chapman

**Affiliations:** 1 Division of Pulmonary and Critical Care Medicine, Medical University of South Carolina, Charleston, SC, USA; 2 Irish Centre for Genetic Lung Disease, Beaumont Hospital, Royal College of Surgeons in Ireland, Dublin, Ireland; 3 Department of Medicine, Pulmonary and Critical Care Medicine, University Medical Center Giessen and Marburg, Philipps-University Marburg, Member of the German Center for Lung Research (DZL), Marburg, Germany; 4 M.A.R.C.O. GmbH & Co. KG Institute for Clinical Research and Statistics, Düsseldorf, Germany; 5 Global Medical Affairs Respiratory, CSL Behring, Marburg, Germany; 6 Biostatistics, CSL Behring, King of Prussia, PA, USA; 7 Clinical Research and Development, CSL Behring, Bern, Switzerland; 8 Conresp, Loerzweiler, Germany; 9 Department of Medicine, University of Toronto, Toronto, ON, Canada

## Abstract

**Background:**

Acute exacerbations of COPD (AECOPD) have unclear impacts on emphysema measurement using computed tomography (CT)-derived 15th percentile lung density (PD15). The aim of this study was to assess the influence of AECOPD on PD15 lung density in α_1_-antitrypsin deficiency.

**Methods:**

In a *post hoc* analysi*s* of the RAPID (Randomised Trial of Augmentation Therapy in α_1_-Proteinase Inhibitor Deficiency) trial, raw marginal residuals of PD15 (measured − predicted) were determined by fitting a regression line to individual patient CT data. These deviations from the expected slope were compared by age, sex, baseline forced expiratory volume in 1 s, diffusing capacity of the lungs for carbon monoxide % predicted and PD15, inhaled corticosteroid use and treatment group.

**Results:**

Positive and negative residuals (reflecting higher or lower lung density than predicted from regression) were observed, which declined in magnitude over time following AECOPD events. Logistic regression confirmed a limited effect of patient characteristics on the absolute size of residuals, whereas AECOPD within 6 weeks of CT had a notable effect *versus* no AECOPD within 6 weeks (OR 5.707, 95% CI 3.375–9.652; p<0.0001).

**Conclusion:**

AECOPD result in higher or lower CT lung density estimates; the effect is greatest in the 2 weeks immediately after an AECOPD and persists for <6 weeks. Patient characteristics were less relevant than AECOPD within 6 weeks, supporting the reliability of PD15 as a measure of lung density. An exacerbation-free period prior to CT scan is advisable to reduce signal-to-noise ratio in future clinical trials.

## Abstract 2

**AECOPD can affect CT lung density estimates in individual patients. The effect is greatest in the first 2** **weeks after AECOPD and declines over time. An AECOPD-free period prior to CT scan is advisable to reduce signal-to-noise ratio in future trials.** https://bit.ly/3XHoMae

## Introduction

α_1_-antitrypsin deficiency (AATD) is associated with an increased risk of early-onset emphysema [1]. AAT protects the lung parenchyma against protease activity, and supplementation of AAT by intravenous infusion is currently the only approved specific treatment for AATD [1, 2]. The efficacy of AAT supplementation may be determined by the change in the trajectory of emphysema progression, which can be quantified by change in lung density [3]. The RAPID (Randomised Trial of Augmentation Therapy in α_1_-Proteinase Inhibitor Deficiency) randomised controlled trial (RCT) was a double-blind, placebo-controlled trial whereby 180 patients with AATD received AAT therapy (Respreeza^®^/Zemaira^®^) or placebo for 2 years [4]. The RAPID open label extension (OLE) study followed 140 of these patients for a further 2 years, with all patients receiving AAT therapy. The RAPID trials utilised computed tomography (CT)-derived 15th percentile lung density (PD15) to quantify change in emphysema progression [4, 5]. In RAPID-RCT, AAT therapy reduced the loss of lung density *versus* placebo by 34% [4], and in RAPID-OLE slowed disease progression when treatment with AAT was started early or late in the study [4, 5]. In RAPID-OLE, the rate of lung density decline was maintained in those who received active treatment in RAPID-RCT (early-start patients), with an inflection seen in the rate of decline temporal to the switch to active treatment in those who received placebo during the double-blind portion of the RAPID trial (delayed-start patients) [5]. These findings, coupled with the observation that delayed-start patients did not “catch up” in terms of lung density loss demonstrated the value of early intervention and a disease-modifying effect of treatment [6].

PD15 is a validated method for quantifying emphysema in AATD [7], and correlates with quality of life and lung function measurements in patients with AATD [7, 8]. However, lung function tests may not detect emphysema until the disease is moderate to severe, when up to 30% of lung parenchyma has been lost [9], suggesting that lung CT-derived lung density loss is a more sensitive measure of emphysema progression in patients with AATD as has previously been demonstrated [10].

Although the impact of acute exacerbations on pulmonary function measurements has been well described [11–13], their impact on CT lung density has not been extensively studied. There is some evidence that an acute exacerbation of COPD (AECOPD) could temporarily induce changes that could alter lung density estimates [14, 15].

Some clinicians suggest that a 6-week exacerbation-free period should be utilised when acquiring CT density measurements [16]; however, to date, no clinical study has determined the optimal length of the exacerbation-free period. Therefore, a *post hoc* analysis of the RAPID trial programme was performed to determine whether exacerbations affect the reliability of CT scan lung density estimation, and to evaluate the influence of patient characteristics including age, sex, baseline lung function, baseline PD15, inhaled corticosteroid (ICS) use and AAT augmentation.

## Study design and methods

### RAPID trial programme

The detailed protocol and results of the RAPID trial programme have been described previously [4, 5]. Briefly, the RAPID trial programme consisted of the 2-year randomised, double-blind placebo-controlled trial (RAPID-RCT) and the 2-year open label extension (RAPID-OLE). For the purpose of this analysis, data were used from the RAPID-RCT trial only, which evaluated the efficacy of 60 mg·kg^−1^ per week *i.v.* infusion of AAT *versus* placebo over 2 years (n=180). Spiral CT scans at total lung capacity were performed at baseline and at 3, 12, 21 and 24 months. Data on AECOPD were derived through a combination of adverse event reporting and diary cards recording symptoms (cough, sputum production and breathlessness), which were collected continuously throughout the study. Exacerbations were defined according to Anthonisen criteria [17].

### Estimation of raw marginal residuals

**FIGURE 1:**
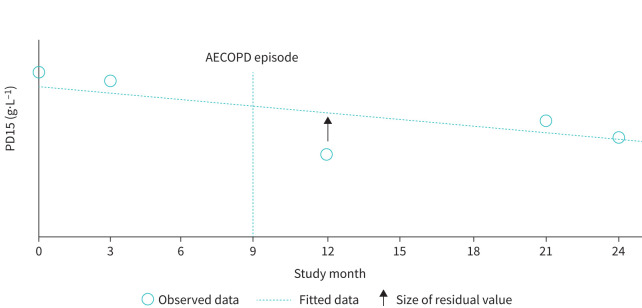
Calculation of individual patient raw marginal residuals for 15th percentile lung density (PD15) measurements. Raw marginal residuals were calculated as the difference between the observed data and the predicted value from the regression line. This deviation from the expected PD15 slope can be above (positive value, denser lungs) or below (negative value, more lucent lungs) the line of regression. AECOPD: acute exacerbation of COPD.

The impact of an exacerbation on lung density estimates from CT scans and their temporal relationship with documented exacerbations were analysed. For each participant, a regression line for PD15 over time was calculated for CT scans for which no AECOPD was documented within the 6 weeks prior to the measurement. Participants with three or more scans were included in the analysis. This regression line can be interpreted as the expected course of PD15 in the absence of AECOPD. Raw marginal residuals were calculated as the difference between the observed data for measurements with or without prior AECOPD and the predicted value from the regression line (figure 1). If AECOPD influence the PD15 measurement, the distribution of the residuals from measurements with prior AECOPD should differ from those measurements without AECOPD events within 6 weeks.

### Association between residuals and patient characteristics

Residual PD15 values were compared based on age, sex, baseline forced expiratory volume in 1 s (FEV_1_) % predicted, baseline diffusing capacity of the lungs for carbon monoxide (*D*_LCO_) % pred, baseline PD15 density, use of ICS within 6 weeks prior to measurement, and treatment group (AAT (Zemaira/Respreeza; CSL Behring, Kankakee, IL, USA) or placebo). “High” and “low” baseline characteristic groups were determined using study population medians of 47.3 g·L^−1^ for PD15, 45.3% pred for FEV_1_, 46.2% pred for *D*_LCO_ and 53 years for age. Analyses were performed four times by graphical and categorical analyses, using all observations in patients who experienced no exacerbations within the past 6 weeks, and in those who experienced exacerbations within the past 2, 4 and 6 weeks.

Scatterplots with regression lines were used to analyse numerical patient characteristics, including Pearson's correlation coefficient and p-values. Histograms and density estimates were used to display categorical patient characteristics, with p-values calculated using the Wilcoxon rank sum test. Results were considered statistically significant when p<0.05; however, no multiplicity adjustment was conducted considering the exploratory nature of the analyses. Scatterplots were generated for residual PD15 values *versus* days since last exacerbation with a penalised B-spline [18].

Raw marginal residuals were grouped into large and small categories, for data with actual lung density values ≥2 g·L^−1^ lower or higher than the predicted value. The 2 g·L^−1^ threshold was chosen to be approximately one standard deviation of the residuals in the population without acute COPD exacerbations (sd=1.7). Absolute and relative frequencies of residual categories by groups of patient characteristics were presented. For numerical characteristics, high and low categories were determined according to the median of the characteristic in the complete analysis population.

A stepwise logistic regression analysis was conducted to determine which parameters influenced the frequency of large residuals >2 g·L^−1^. Numerical values were used for numerical patient characteristics, and in addition to characteristics listed above, “exacerbation within the last 6 weeks (yes/no)” was also included as a possible independent variable. Stepwise regression began as an empty model, with independent variables added and removed from the model applying a p-value of 0.2. p-values were generated using the Wald test; p-values <0.2 were considered possible parameters of influence. All analyses were conducted using SAS, version 9.4.

## Results

### Calculation of residuals and effect of prior AECOPD

**FIGURE 2:**
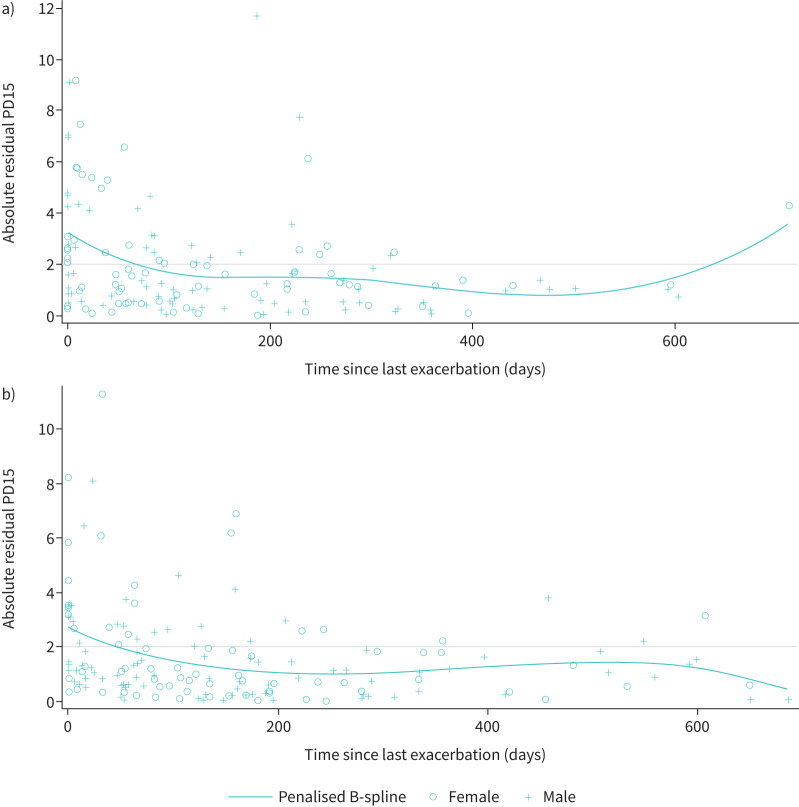
Raw marginal residual 15th percentile lung density (PD15) values against time since last exacerbation. a) Positive and b) negative raw marginal residual PD15 values and spline by time since last exacerbation. Measurements are from scans with at least one prior exacerbation.

Of the 180 patients in the RAPID-RCT, 132 patients had three or more valid CT scans without an AECOPD within the 6 weeks prior to lung density quantification. These patients had a total of 633 scans. Patients with an exacerbation in the past 6 weeks had higher residuals than patients without an exacerbation in the past 6 weeks. Standard deviations were larger for residual PD15 values at time points closer to the occurrence of an AECOPD, and appeared to decrease in magnitude between 2 and 6 weeks (figure 2). Exacerbations resulted in a greater frequency of large residuals (2 g·L^−1^ higher or lower than the observed value) *versus* no exacerbations: 56.6% of 76 measurements with AECOPD ≤6 weeks from CT had large residuals *versus* 19.4% of 557 measurements with no AECOPD (p<0.0001; table 1).

**TABLE 1:** Descriptive analysis of the association between patient characteristics and residuals

	**Total measurements**	**Residual** **inside** **(−2–2 g·L^−1^)**	**Residual outside** **(−2–2 g·L^−1^)**	**p-value**	**Residual** **≤−2 g·L^−1^**	**Residual** **>2 g·L^−1^**
**No AECOPD within 6 weeks**						
All measurements	557	449 (80.6)	108 (19.4)		53 (9.5)	55 (9.9)
AAT treatment group	285	222 (77.9)	63 (22.1)		32 (11.2)	31 (10.9)
Placebo group	272	227 (83.5)	45 (16.5)	0.1081	21 (7.7)	24 (8.8)
Age at baseline <53 years	264	219 (83.0)	45 (17.0)		22 (8.3)	23 (8.7)
Age at baseline ≥53 years	293	230 (78.5)	63 (21.5)	0.1987	31 (10.6)	32 (10.9)
Female patients	224	166 (74.1)	58 (25.9)		27 (12.1)	31 (13.8)
Male patients	333	283 (85.0)	50 (15.0)	**0.0021**	26 (7.8)	24 (7.2)
Baseline FEV_1_ <45.3%	274	226 (82.5)	48 (17.5)		26 (9.5)	22 (8.0)
Baseline FEV_1_ ≥45.3%	283	223 (78.8)	60 (21.2)	0.2853	27 (9.5)	33 (11.7)
Baseline *D*_LCO_ <46.2%	274	228 (83.2)	46 (16.8)		23 (8.4)	23 (8.4)
Baseline *D*_LCO_ ≥46.2%	273	212 (77.7)	61 (22.3)	0.1069	30 (11.0)	31 (11.4)
Baseline PD15 <47.3	269	231 (85.9)	38 (14.1)		18 (6.7)	20 (7.4)
Baseline PD15 ≥47.3	281	212 (75.4)	69 (24.6)	**0.0025**	35 (12.5)	34 (12.1)
ICS	465	374 (80.4)	91 (19.6)		44 (9.5)	47 (10.1)
No ICS	92	75 (81.5)	17 (18.5)	0.8859	9 (9.8)	8 (8.7)
**AECOPD within 6 weeks**						
All measurements	76	33 (43.4)	43 (56.6)		18 (23.7)	25 (32.9)
AAT treatment group	43	14 (32.6)	29 (67.4)		11 (25.6)	18 (41.9)
Placebo group	33	19 (57.6)	14 (42.4)	**0.0371**	7 (21.2)	7 (21.2)
Age at baseline <53 years	37	16 (43.2)	21 (56.8)		9 (24.3)	12 (32.4)
Age at baseline ≥53 years	39	17 (43.6)	22 (56.4)	1.0000	9 (23.1)	13 (33.3)
Female patients	37	12 (32.4)	25 (67.6)		10 (27.0)	15 (40.5)
Male patients	39	21 (53.8)	18 (46.2)	0.0687	8 (20.5)	10 (25.6)
Baseline FEV_1_ <45.3%	42	17 (40.5)	25 (59.5)		12 (28.6)	13 (31.0)
Baseline FEV_1_ ≥45.3%	34	16 (47.1)	18 (52.9)	0.6443	6 (17.6)	12 (35.3)
Baseline *D*_LCO_ <46.2%	38	18 (47.4)	20 (52.6)		8 (21.1)	12 (31.6)
Baseline *D*_LCO_ ≥46.2%	38	15 (39.5)	23 (60.5)	0.6438	10 (26.3)	13 (34.2)
Baseline PD15 <47.3	41	20 (48.8)	21 (51.2)		8 (19.5)	13 (31.7)
Baseline PD15 ≥47.3	35	13 (37.1)	22 (62.9)	0.3581	10 (28.6)	12 (34.3)
ICS	66	28 (42.4)	38 (57.6)		17 (25.8)	21 (31.8)
No ICS	10	5 (50.0)	5 (50.0)	0.7386	1 (10.0)	4 (40.0)

Data are presented as n or n (%), unless otherwise stated. For numerical variables, all patients with modelled residuals have been grouped according to their median. p-values were generated using Fisher's exact test for association between the variable and residual inside or outside −2–2 g·L^−1^. Bold values are those with a p-value <0.05. AECOPD: acute exacerbation of COPD; AAT: α_1_-antitrypsin; FEV_1_: forced expiratory volume in 1 s; *D*_LCO_: diffusing capacity of the lungs for carbon monoxide; PD15: 15th percentile lung density; ICS: inhaled corticosteroids.

### Effect of patient baseline parameters on residuals

**FIGURE 3:**
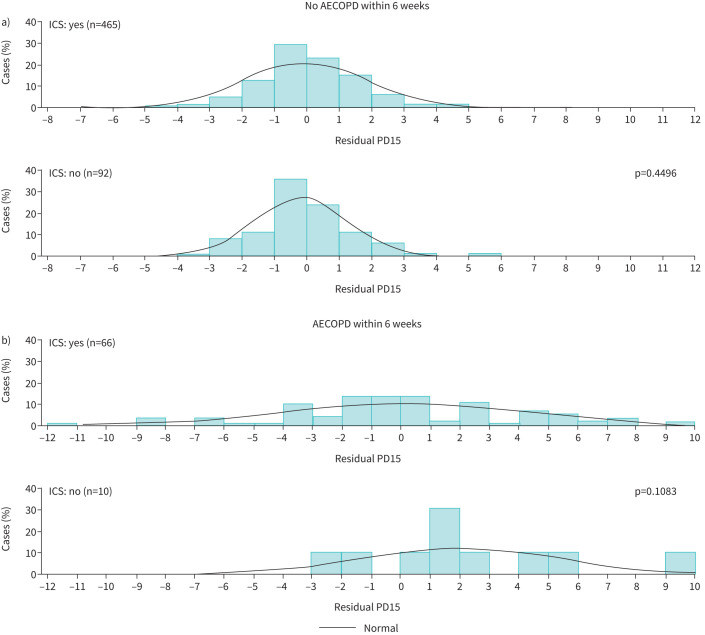
Effect of inhaled corticosteroid (ICS) use on raw marginal residual 15th percentile lung density (PD15) values. p-values were calculated using Wilcoxon's rank-sum test, comparing residuals from patients receiving inhaled corticosteroids and untreated patients a) without acute exacerbation of COPD (AECOPD) and b) following AECOPD.

There were more positive residual PD15 lung density values in relation to AECOPD within 6 weeks without associated ICS use relative to those with ICS use. However, this did not reach statistical significance, possibly due to the small number of patients in this subgroup (n=10, p=0.108; figure 3b). There were few other notable observations arising from the graphical analysis (supplementary figures e2–e7), and overall, none of the patient characteristics analysed resulted in different mean residual PD15 values. The trend line of PD15 by age (supplementary figure e4) shows a slight downward sloping of PD15 in patients with exacerbations compared to a flatter curve in those without exacerbations in RAPID-RCT. This might correlate to more rapid emphysema progression in AATD when exacerbations are present, as occurs in usual COPD.

For all CT scans that were obtained with no AECOPD within the past 6 weeks, the size of the residuals was within ±2 g·L^−1^ in ∼80% of the cases; large residuals were detected in only 20% (table 1). The distribution of residuals was similar following the categorical analysis of various parameters, with two exceptions: females displayed large residuals more frequently than males (25.9% *versus* 15.0%, p=0.002), and large residuals were more frequent in observations from patients who had a higher baseline PD15 lung density (24.6% *versus* 14.1%, p=0.003; table 1).

For CT scan data obtained within 6 weeks of an AECOPD, the distribution was reversed, with more residuals outside the ±2 g·L^−1^ bracket than inside it (56.6% *versus* 43.4%; table 1). Again, females had a greater proportion of large residuals compared to males; however, this was not statistically significant (possibly due to reduced sample size; table 1). There was a notable difference between high and low baseline PD15 following AECOPD, although there was no statistical significance (table 1).

With regard to the study drug intervention, large residuals were more frequent in observations associated with the use of AAT *versus* use of placebo at all time points, with statistical significance reached in observations with an AECOPD within the past 6 weeks (p=0.037; table 1). In addition, the AAT treatment group displayed a greater frequency of larger residuals that were in the positive direction, rather than negative (41.9% *versus* 25.6%). No significant relationships were found between large PD15 residuals and age, ICS use, baseline FEV_1_ % pred or baseline *D*_LCO_ % pred (table 1).

### Logistic regression analysis

Since there were multiple characteristics associated with increased frequency of large PD15 residuals, a stepwise logistic regression analysis was performed on all data, regardless of AECOPD presence and timing, to assess which parameters have the greatest influence on large PD15 residuals. The most influential parameter for large residuals (at least ±2 g·L^−1^) was the presence of AECOPD within the past 6 weeks (OR 5.707, 95% CI 3.375–9.652; p<0.0001; table 2). In addition, high baseline PD15 lung density, female sex and AAT treatment had a greater chance of displaying large residuals than their respective counterparts (table 2).

**TABLE 2:** Summary of logistic regression for parameters that affect raw marginal residuals of 15th percentile lung density (PD15)

	**Estimate OR (95% CI)**	**p-value (Wald)**
**Baseline PD15**	1.030 (1.017–1.044)	<0.0001
**Exacerbation within past 6 weeks: yes *versus* no**	5.707 (3.375–9.652)	<0.0001
**Sex: female *versus* male**	1.765 (1.181–2.636)	0.0055
**Treatment group: AAT *versus* placebo**	1.736 (1.159–2.600)	0.0074

AAT: α_1_-antitrypsin.

## Discussion

The analyses revealed that an AECOPD within 6 weeks of a CT scan may result in greater signal-to-noise ratio (seen as higher or lower lung density readings), with the effect most pronounced within 2 weeks of the CT scan. This could reflect an exacerbation-induced increase in lung tissue density by inflammation (*e.g.* oedema and/or cellular infiltrate), mucus or bacterial load, leading to a temporal increase of lung density. Conversely, a temporal increase in airway obstruction could lead to hyperinflation and a decrease of lung density.

While differences from the predicted values were relatively small (typically <2 g·L^−1^), the inclusion of values obtained from patients with an exacerbation introduced a bias in the calculation of the regression line. Clinically, this suggests that omitting data collected within 6 weeks of an AECOPD may enhance the precision of PD15 lung density estimations on emphysema progression; however, future trial designs may choose a shorter window, since most of the larger variation in lung density occurs within 2 weeks of the exacerbation.

Graphical, categorical and regression analyses demonstrated limited effects of patient characteristics on lung density, supporting the reliability of PD15 as a measure of lung density in patients with AATD. Other evidence of the clinical relevance of CT lung density decline is based on long-term correlations with FEV_1_ decline [5, 7, 19, 20].

Patient parameter effects were limited. ICS was associated with a trend towards reduced mean PD15 following an AECOPD, and AAT treatment was associated with increased frequency of large differences in CT following an AECOPD. Due to the small sample size (n=10) of patients with AECOPD not receiving ICS, this result should be interpreted with caution, and more data would be required to confirm the results. High baseline PD15 and female sex were associated with an increased frequency of large differences in CT residuals in the absence of any AECOPD. In the logistic regression analysis, all of these parameters, except ICS treatment, maintained a statistically significant impact on the odds of higher/lower lung density.

The clinical reasons for larger variation in lung density during an exacerbation are speculative. Airways and parenchymal inflammation are prominent in COPD exacerbations, and it is known that AATD patients frequently have wheezing associated with exacerbations [21], a clinical parameter frequently associated with hyperinflation. Further clinical investigations would be required to further elucidate the clinical correlates of these changes in lung density. The reason for the higher frequency of large variation in lung density observed in patients who received AAT treatment is unclear. One hypothesis is that AAT influences the intensity or the resolution of inflammatory changes in the airways or the lung associated with an AECOPD. It is possible that the higher frequency of large residuals in the AAT-treated group is associated with the slightly higher rate of exacerbations observed in the AAT group *versus* the placebo group in the RAPID-RCT (1.70 *versus* 1.42 annually) [4]. Due to the bidirectional nature of the residuals, it is unlikely that the increased residuals in the AAT treatment group are due to increased protein volume in the vasculature following infusion. Furthermore, due to the small magnitude of the changes and bidirectional nature, variation in CT measurements in relation to the study interventions would not be expected to affect the main findings of the RAPID trials.

CT-derived PD15, as used in the RAPID trials, is a validated method for quantifying emphysema in AATD [7], which correlates with lung function and other disease parameters, and has proven more sensitive than other techniques and measurements for measuring or determining emphysema progression in AATD therapy trials [3, 19]. This method of densitometry calculation was the most sensitive index of measuring emphysema progression in a prior AAT therapy trial [22]. Obtaining accurate and precise values from CT scans by minimising factors affecting the measurements is essential in order to determine therapy-related changes in lung density [3, 23]. The effect of exacerbations on CT-derived lung density is not well characterised; however, one study in asthma reported that exacerbations resulted in lower lung density, and that similar to the present results, lung density was changed by corticosteroid use [14].

From our analyses, there is no indication regarding patient characteristics predictive of higher or lower lung density in response to exacerbations, and further analysis is required to determine if patient baseline parameters are associated with the direction of the lung density variation (*i.e.* positive or negative). In agreement with the present study, sex differences in lung density, and the rate of its decline, have been reported previously in COPD [24] and lung cancer [25], possibly since the adverse effects of smoking on lung function may affect females to a greater extent than males [26]. Other work has hypothesised that the variation may be due to anatomical differences, with females having more lung tissue per volume than males [27]. Since oestrogen affects plasma volume [28] and is variable in females, the variability in lung density may be partly oestrogen-related. The influence of sex on lung density requires additional study. Besides smoking status, prior studies have also reported little effect of patient parameters on lung density [14, 25].

Although CT densitometry of the lung is not widely used in clinical evaluation, trials of AAT therapy have shown that PD15 lung density is a reliable end-point for quantifying emphysema progression, and was the only method sensitive enough to detect a treatment effect [4, 5, 19, 29]. This *post hoc* analysis supports this concept, in so far as PD15 demonstrated limited variability in relation to patient characteristics.

Despite the known reliability of lung density, standardisation of algorithms used by different CT scanners and scanning protocols is an aspect currently lacking; efforts are being made to determine and standardise for the technological factors affecting the reliability and sensitivity of CT densitometry [30, 31]. The present analysis suggests that standardisation efforts should include considerations regarding the effect of exacerbations. In particular, it is known that exacerbations in AATD are associated with higher levels of inflammation *versus* general COPD [32]; therefore, a defined robust exacerbation-free period prior to CT lung density acquisition is critical to minimise variability in the results of future research. A reduced signal-to-noise ratio may result in more efficient trials, with fewer study participants required.

The present analysis supports the adequacy of a 6-week exacerbation-free period; however, some limitations of the present analysis should be noted. Firstly, the sample size of CT scans that took place within 6 weeks of an AECOPD was relatively small (n=76), which could account for a lack of statistical power in these analyses. Secondly, since factors that simultaneously cause positive (*e.g.* inflammation) and negative (*e.g.* air-trapping) signals in the same CT examination may result in regression to the mean; these effects at the time of exacerbations could be more frequent than currently recognised.

### Conclusion

This study suggests that AECOPDs can affect PD15 densitometry values; however, the effect is relatively small and highest in the first 2 weeks following an AECOPD, and the influence of patient baseline parameters is equally minimal. A 6-week post-exacerbation period would represent a conservative approach to obtain reliable data for determining emphysema progression in future clinical trials to minimise variability in CT lung density outcomes where the number of patients available for study remains restricted by the rarity of the disease.

## Supplementary material

10.1183/23120541.00457-2022.Supp1 **Please note:** supplementary material is not edited by the Editorial Office, and is uploaded as it has been supplied by the author.
Supplementary material 00457-2022.SUPPLEMENT
