# Supplementary material for: The effect of exacerbations on lung density in α1-antitrypsin deficiency
Source: ERJ Open Res. 2023 Mar 13;9(2):00457-2022. doi: 10.1183/23120541.00457-2022 (PMC10009703; doi:10.1183/23120541.00457-2022)
Supplement: Supplementary file 1 [file 00457-2022.SUPPLEMENT.pdf]

Supplementary material

eFigure 1: **Density of raw marginal residuals**

Residual PD15 values with no AECOPD within 6 weeks (top) and AECOPD within 6 weeks (bottom).

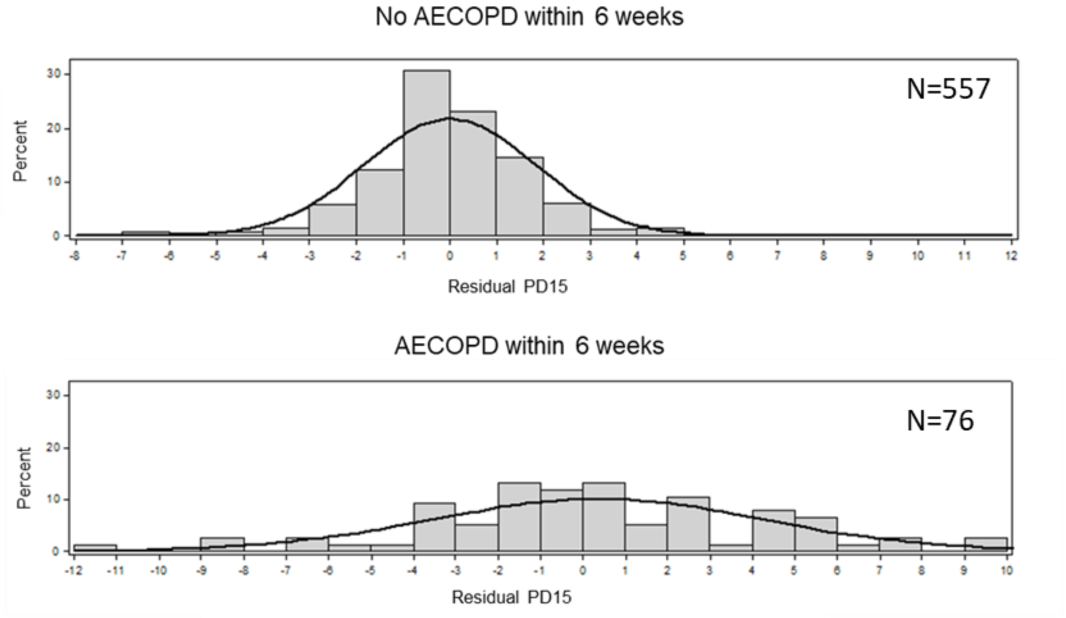

AECOPD, acute exacerbation of chronic obstructive pulmonary disease; PD15, 15th percentile lung density.

eFigure 2: Density of raw marginal residuals by treatment group

PD15 values with no AECOPD within 6 weeks (A) and AECOPD within 6 weeks (B) by treatment group.

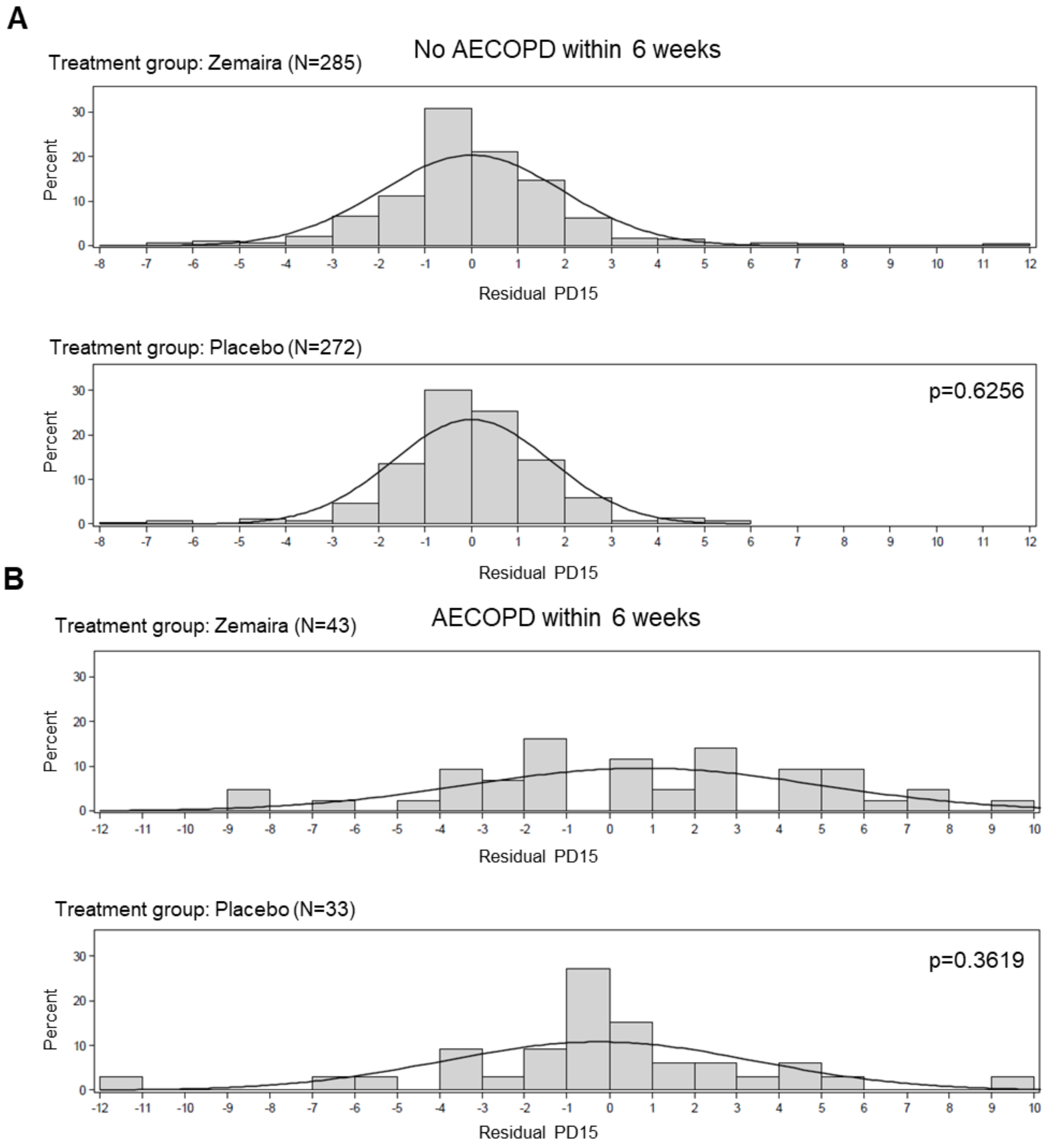

AECOPD, acute exacerbations of chronic obstructive pulmonary disease; PD15, 15th percentile lung density.

eFigure3: Density of raw marginal residuals by sex

PD15 values with no AECOPD within 6 weeks (A) and AECOPD within 6 weeks (B) by sex.

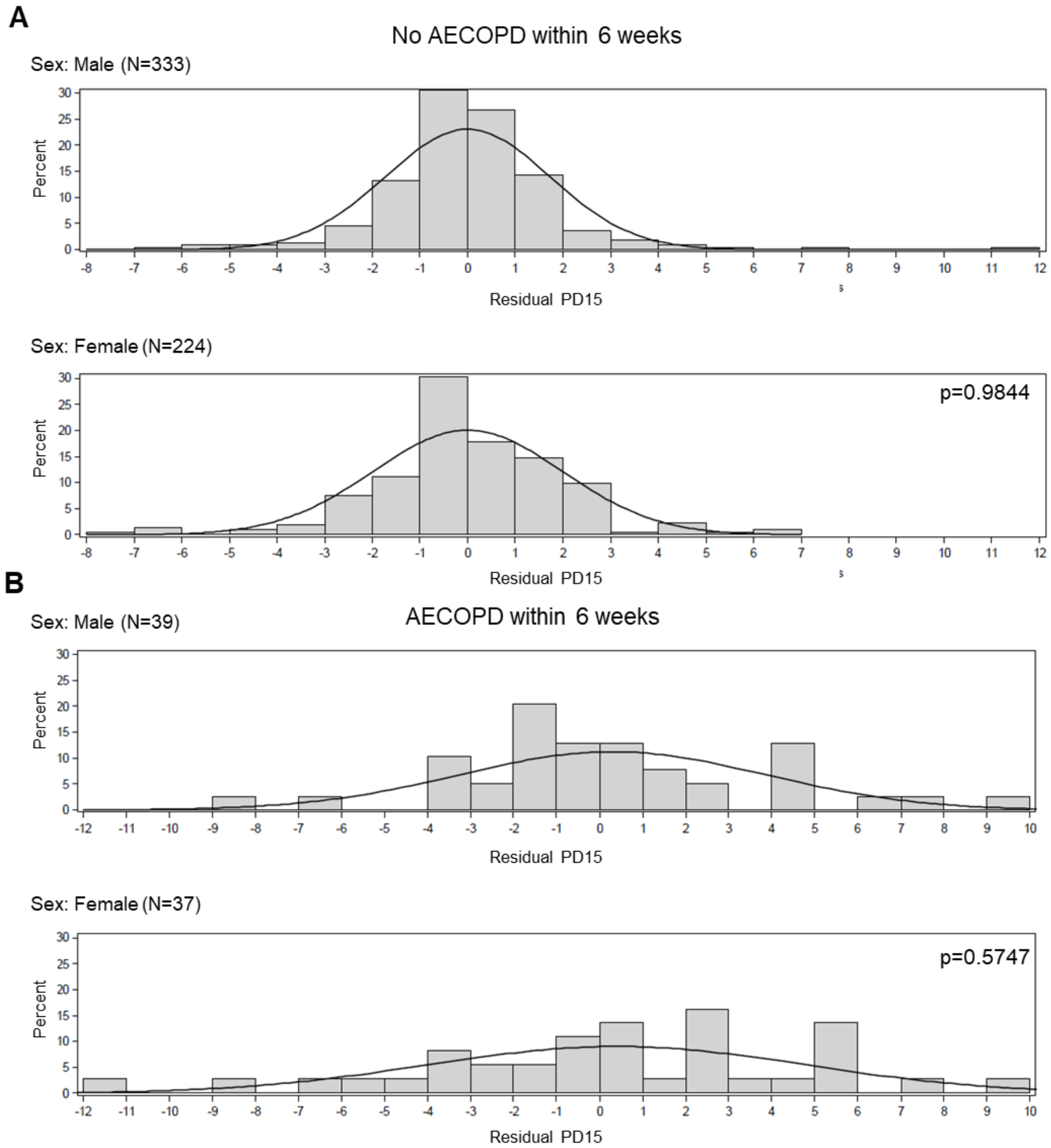

AECOPD, acute exacerbations of chronic obstructive pulmonary disease; PD15, 15th percentile lung density.

eFigure 4: Scatterplot of raw marginal residuals by age

PD15 values with no AECOPD within 6 weeks (A) and AECOPD within 6 weeks (B) *vs*. age.

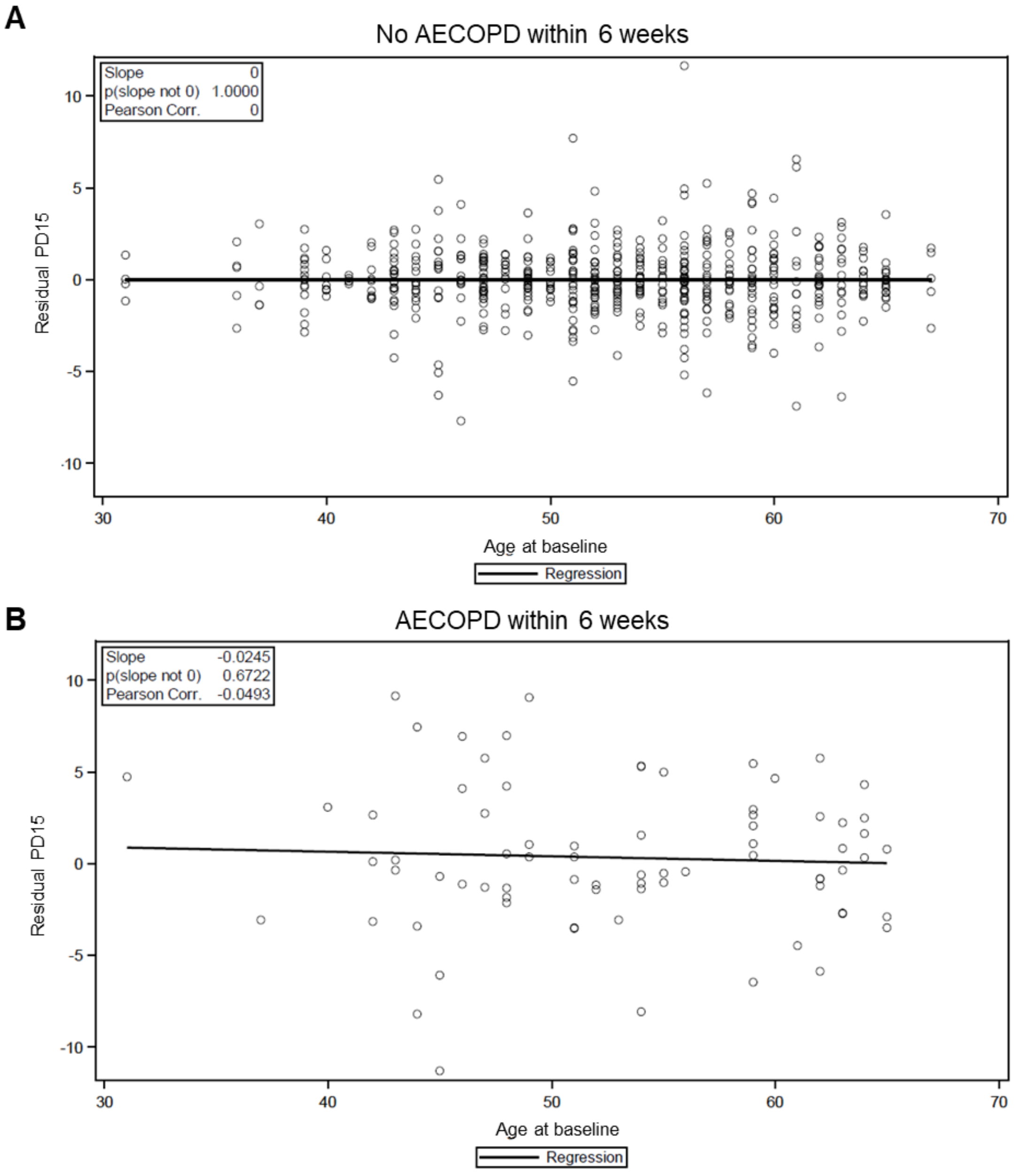

AECOPD, acute exacerbations of chronic obstructive pulmonary disease; PD15, 15th percentile lung density.

eFigure 5: Scatterplot of raw marginal residuals by baseline FEV_1% predicted

PD15 values with no AECOPD within 6 weeks (A) and AECOPD within 6 weeks (B) *vs*. baseline FEV_1 % predicted.

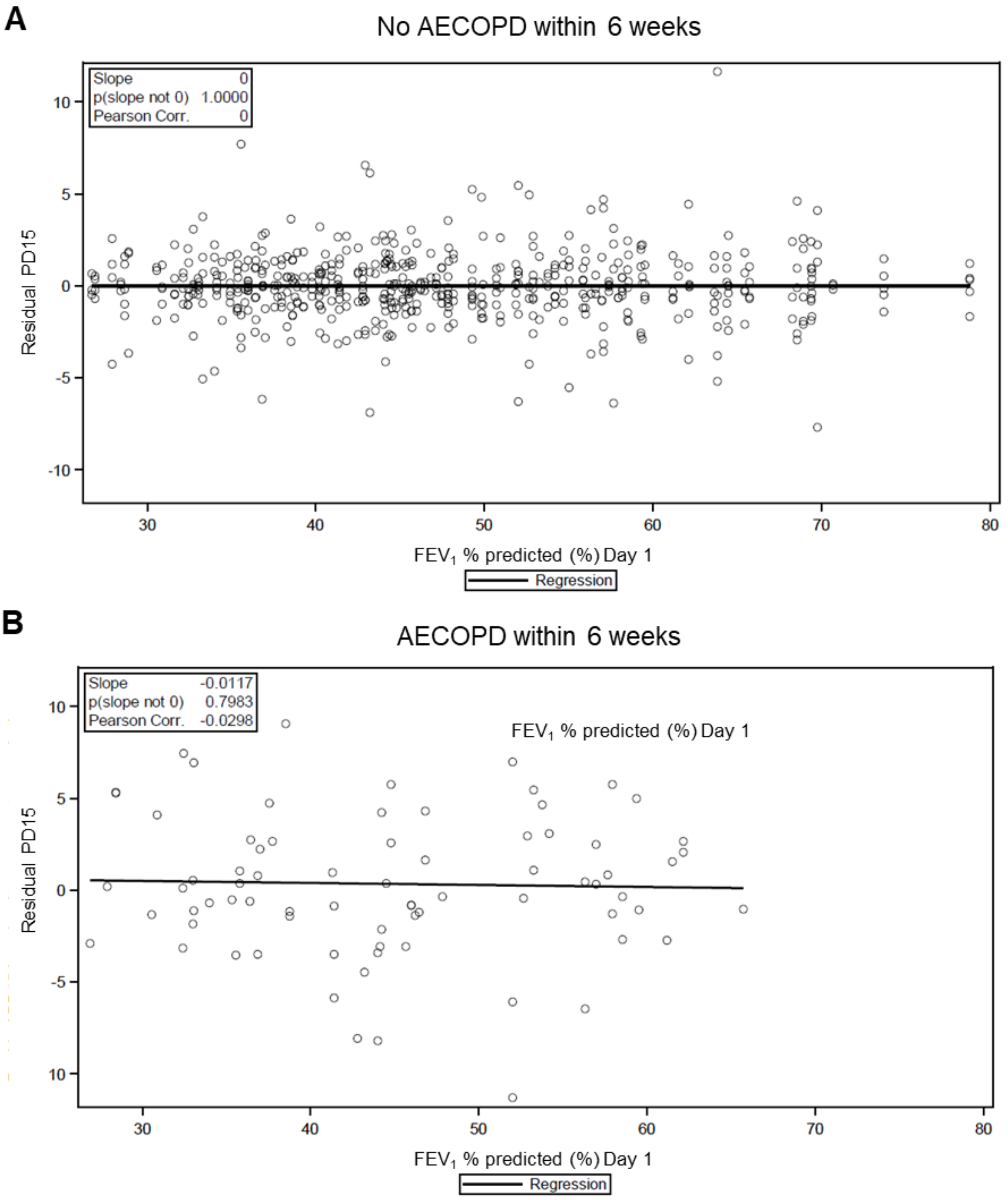

AECOPD, acute exacerbation of chronic obstructive pulmonary disease; FEV_1, forced expiratory volume in one second; PD15, 15th percentile lung density.

eFigure 6: Scatterplot of raw marginal residuals by baseline DL_{CO}% predicted

PD15 values with no AECOPD within 6 weeks (A) and AECOPD within 6 weeks (B) *vs*. baseline DL_{CO}% predicted.

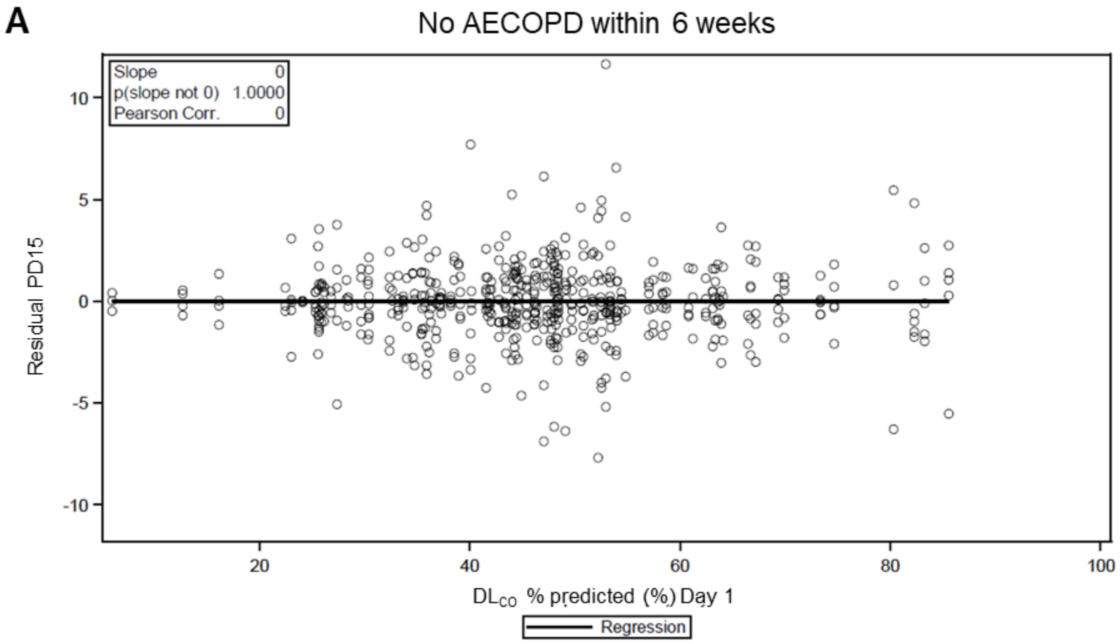

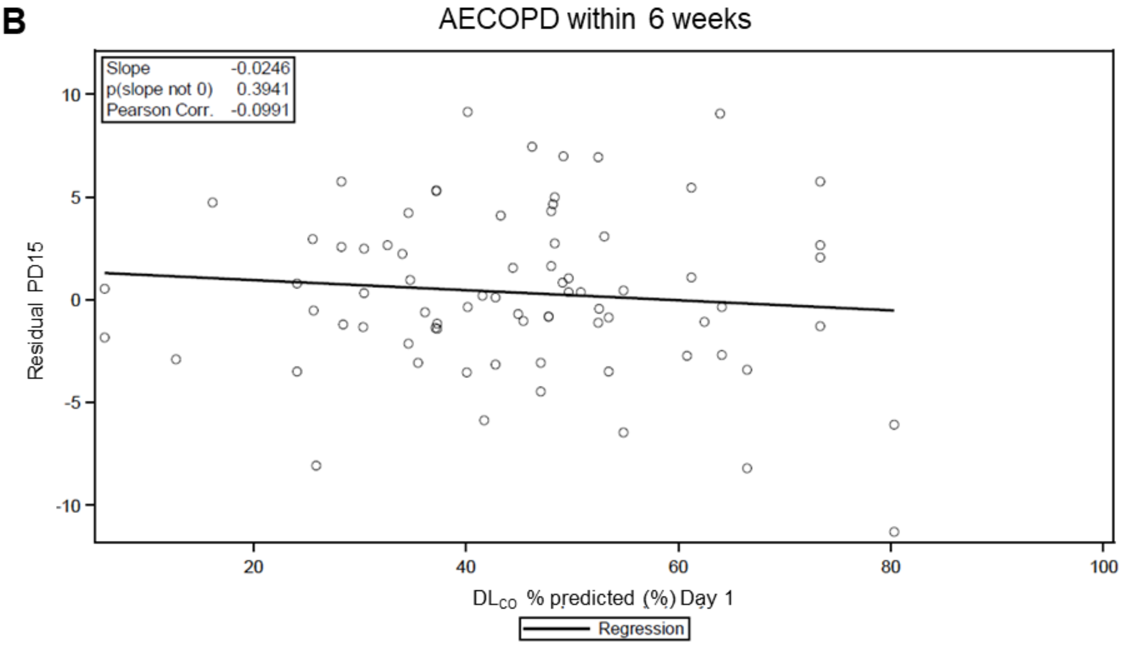

AECOPD, acute exacerbations of chronic obstructive pulmonary disease; DL_{co}, diffusing capacity of the lungs for carbon monoxide; PD15, 15th percentile lung density.

eFigure 7: Scatterplot of raw marginal residuals by baseline PD15

PD15 values with no AECOPD within 6 weeks (A) and AECOPD within 6 weeks (B) *vs*. baseline PD15.

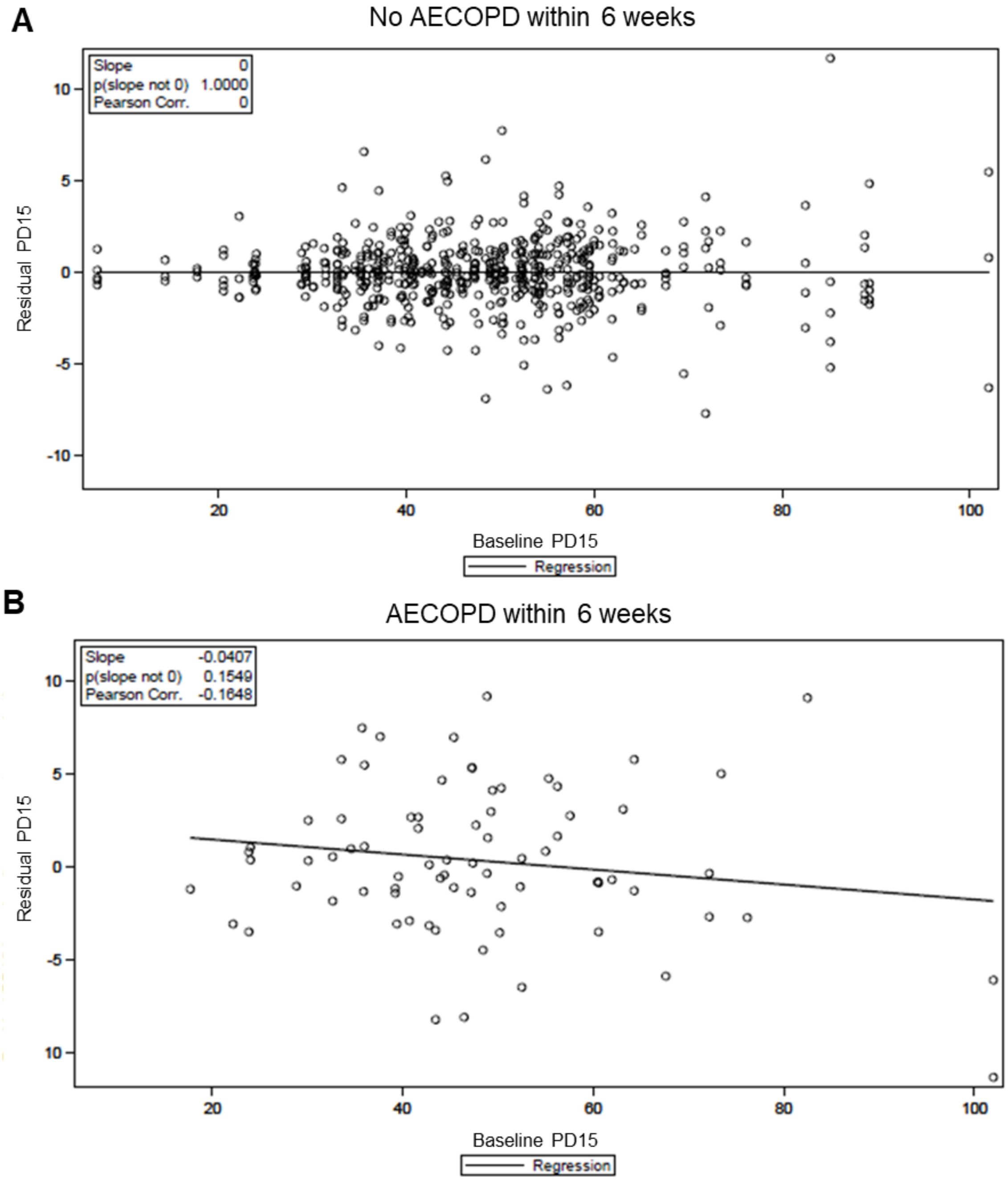

AECOPD, acute exacerbations of chronic obstructive pulmonary disease; PD15, 15th percentile lung density.
